# The Prevention of Disease

**Published:** 1894-06

**Authors:** 


### The Prevention of Disease.

A paper on this subject was read before the New York Academy of Medicine at its general meeting, Feb. 1, 1894, by W. W. Potter, of Buffalo, N. Y. He said:

The highest office of the physician of modern times is, and ever must be, in the exercise of his functions toward the prevention of disease. Every one is, or should be, interested in this subject.

The physician must be prepared to offer an intelligent opinion as to the best methods of preventing the spread of cholera, small-pox, etc., etc., as well as on questions pertaining to plumbing, sewerage, drainage, ventilation, and every detail of house sanitation.

Happily, however, some of the exanthematous fevers have not now the same dread as formerly, since the triumph in medicine in solving the methods of their prevention and cure. But a number of maladies, that formerly were not thought to be contagious or infectious, are now known to be so through the investigations of clinicians and bacteriologists.

Taking as an example consumption. While it is true that much has been done of late to control its spread, yet how many physicians are ready to admit that it is an infectious malady, and that it may be conveyed to a healthy person?

In order to prevent the spread of this direful disease effectively, it would be necessary to establish an absolute inspection over all milch cows, their feed and stabling and over all animals that are slaughtered for the purposes of food, to the end that every tuberculous beast shall be killed and its body cremated.

The quantities of tuberculous food consumed are enormous, and it will require the most rigid system of inspection and supervision by most skilled, honest and well-paid officials to stop traffic in these dangerous and unwholesome aliments.

Another important step toward the prevention of the propagation of consumption consists in the prohibition of the intermarriage of tuberculous subjects; for a man with existing tubercular disease to marry a woman of tubercular parentage would be tempting providence. The off-spring of such a marriage, to say the least, would offer a favorable soil for the growth of the tubercle bacillus. Still another step in the direction of prevention consists in the establishment of separate hospitals for consumptives, and the adoption of measures looking toward their isolation. The sputum of the consumptive constitutes a focus of special danger. To deal intelligently with the question of prevention the sputum must be sterilized as soon as thrown off.

Without doubt, independent of the infection by food, the most serious menace comes from the respiration of infected dust, and the one great means of infecting dust is through the sputum of consumptives. The author is of the opinion that it will be yet just as possible to quarantine and control the method and habits of life of the consumptive patient as it is now of a small-pox, yellow fever, or cholera patient. At all events, he hopes that an enlightenment of the people on this subject will lead to such results.—*American Medico-Surgical Bulletin.*

